# The Construction and Comprehensive Prognostic Analysis of the LncRNA-Associated Competitive Endogenous RNAs Network in Colorectal Cancer

**DOI:** 10.3389/fgene.2020.00583

**Published:** 2020-06-23

**Authors:** Wei Li, Weifang Yu, Xia Jiang, Xian Gao, Guiqi Wang, Xiaojing Jin, Zengren Zhao, Yuegeng Liu

**Affiliations:** ^1^Department of General Surgery, Hebei Key Laboratory of Colorectal Cancer Precision Diagnosis and Treatment, The First Hospital of Hebei Medical University, Shijiazhuang, China; ^2^Departments of Endoscopy Center, The First Hospital of Hebei Medical University, Shijiazhuang, China; ^3^Department of Emergency, The First Hospital of Hebei Medical University, Shijiazhuang, China

**Keywords:** colorectal cancer, differentially expressed RNAs, ceRNA network, overall survival, nomogram

## Abstract

Competing endogenous RNAs (ceRNAs) are a newly proposed RNA interaction mechanism that has been associated with the tumorigenesis, metastasis, diagnosis, and predicting survival of various cancers. In this study, we constructed a ceRNA network in colorectal cancer (CRC). Then, we sought to develop and validate a composite clinicopathologic–genomic nomogram using The Cancer Genome Atlas (TCGA) database. To construct the ceRNA network in CRC, we analyzed the mRNAseq, miRNAseq data, and clinical information from TCGA database. LncRNA, miRNA, and mRNA signatures were identified to construct risk score as independent indicators of the prognostic value in CRC patients. A composite clinicopathologic–genomic nomogram was developed to predict the overall survival (OS). One hundred sixty-one CRC-specific lncRNAs, 97 miRNAs, and 161 mRNAs were identified to construct the ceRNA network. Multivariate Cox proportional hazards regression analysis indicated that nine-lncRNA signatures, eight-miRNA signatures, and five-mRNA signatures showed a significant prognostic value for CRC. Furthermore, a clinicopathologic–genomic nomogram was constructed in the primary cohort, which performed well in both the primary and validation sets. This study presents a nomogram that incorporates the CRC-specific ceRNA expression profile, clinical features, and pathological factors, which demonstrate its excellent differentiation and risk stratification in predicting OS in CRC patients.

## Introduction

The third most common type of cancer is colorectal cancer (CRC), which is also ranked as the second most frequent cause of cancer-related death worldwide (Bray et al., 2018). In 2018, more than 1.8 million patients are diagnosed with CRC where at least 881,000 of them die of the complications brought upon by the disease (Bray et al., 2018). The pathogenesis of CRC involves multiple factors, including genetic and environmental factors (Jeon et al., 2018). Colorectal cancer remains a global health concern because it is highly prevalent, and its prognosis remains poor despite the rapid emergence of tools for diagnosis and treatments (Camp and Ellis, 2015; Li et al., 2018). As a result, it is imperative to improve the diagnostic approach, prognostic prediction, and survival outcomes by identifying novel therapeutic targets in CRC.

Medical literature has highlighted the important and active roles of different types of RNAs in the cancer’s progression and development (Lee et al., 2017; Wu et al., 2017). In the human genome sequence, more than 90% could be transcribed but could not code for proteins (Xiao et al., 2016). The majority of the transcriptome is accounted for by non-coding RNAs or ncRNAs. Continuous discovery has emerged on the different regulative RNA classes with vital biological functions (Baltimore, 2001). Non-coding RNAs of more than 200 bp are classified as the long non-coding RNAs (lncRNAs) (St Laurent et al., 2015). Recent research highlights the key functions of lncRNAs in the regulation of biological processes. These include genomic imprinting, immune response, cell cycle and apoptosis, immune response, and different types of cancer (Beermann et al., 2016; Fang and Fullwood, 2016). In CRC, lncRNAs have been known with the possibility to suppress tumors or function as oncogenes (Di et al., 2019). Current research indicates the function of lncRNAs as miRNA sponges and competing endogenous RNAs (ceRNAs). This reduces the availability of miRNAs for mRNA target binding (Salmena et al., 2011; Tay et al., 2014). The ceRNA hypothesis underlines the interaction of the RNA with miRNA response elements (MREs). This type of crosstalk and competition in the RNA also occurs among mRNAs and lncRNAs (Zhang et al., 2016a). Further research is recommended on lncRNAs’ function as ceRNAs. Additionally, studies with larger sample sizes should be conducted on CRC’s association with lncRNA-mediated ceRNA network in a whole-genome sequence.

Nomogram is a statistical prediction model that combines multiple prognostic factors to make intuitive graphical and individualized predictions (Iasonos et al., 2008). Up to date, various kinds of prognostic models were reported in CRC (Tian et al., 2017; Xiong et al., 2018; Zhu et al., 2018). Although doubts about the predictive value of clinicopathological features are increasing, they still provide the most reliable guidelines for the prognostication and treatment of CRC (Zhang et al., 2016b). Thus, we deduced that integrating genomic signatures with clinicopathological features in a model would yield a predictive accuracy exceeding that of the currently available prognostic system. Here, we aimed to apply a systematic approach to evaluate the clinical usefulness of CRC-related signatures and then construct a composite clinicopathologic–genomic nomogram by integrating factors with potential prognostic value based on TCGA database.

## Materials and Methods

### Data Collection

We generated data on CRC from TCGA. Through the GDC apps, we downloaded the gene expression data of the mRNAseq (level 3) through the Data Transfer Tool. Also, we downloaded the clinical information of CRC patients and the samples of miRNAseq^1^. To achieve an accurate and completely annotated lncRNA and mRNA datasets, we compared the level 3 mRNAseq gene expression data with the annotation information of the Ensembl Genome Browser 99(GRCh38.p13) database^2^ (Cunningham et al., 2019). The data used in this study met the following criteria: (a) CRC patients with complete information on age, gender, tumor location, tumor recurrence, neoplasm cancer, residual tumor, tumor stage, T stage, N stage, and M stage; (b) patients with complete lncRNAseq, mRNAseq, and miRNAseq data. Finally, 335 CRC samples and 51 normal control samples were collected in our study. Our research meets the publication guidelines provided by TCGA^3^.

### Differentially Expressed Analysis

We applied the “edgeR” package in R to identify differentially expressed lncRNAs (DElncRNAs), miRNAs (DEmiRNAs), and mRNAs (DEmRNAs) between CRC and adjacent normal tissues (Robinson et al., 2010). Thresholds for the screening of differentially expressed RNAs were adjusted *P* < 0.05 and |log_2_fold change (FC)| > 2.

### Functional Annotation

We performed analyses on the functional and pathway enrichment of DEmRNAs with the use of a R package called “clusterprofiler” (Yu et al., 2012) that includes the pathway enrichment analyses of the Kyoto Encyclopedia of Genes and Genomes (KEGG) (Kanehisa et al., 2004) and the Gene Ontology (GO) (Ashburner et al., 2000). Statistical significance of functional categories, visualized using R, is set at a false discovery rate (FDR) of < 0.05.

### Construct the ceRNA Network

We created a ceRNA network that was built on the hypotheses that the potential impact of lncRNAs on miRNAs is evident, and it could also carry out the behaviors of miRNA sponges for more enhanced regulation of mRNAs. The ENCORI forecasted the lncRNA–miRNA interactions^4^. These interactions were founded on specific CRC miRNAs. Through miRDB^5^, TargetScan^6^, and miRTarBase^7^, our team predicted the miRNA-targeted mRNAs. Only mRNAs recognized by all three databases were considered candidate targets and overlapping with the identified DEmRNAs to screen out the DEmRNAs that were targeted by the DEmiRNAs. A careful cross-matching was executed between the predicted results and the results of the DEmiRNAs, DElncRNAs, and DEmRNAs. We accessed the visual overview of the results through Cytoscape 3.7.0 (Cytoscape Consortium, San Diego, CA, United States) from these networks (Shannon et al., 2003).

### Survival Analysis

With the use of the survival package in R, we plotted the Kaplan–Meier curves of the lncRNAs, miRNAs, and mRNAs in the ceRNA network along with the combination of the CRC patients’ clinical data in TCGA. The optimal cutoff values for RNA expression were determined by X-tile software (version 3.6.1; Yale University, New Haven, CT, United States) (Camp et al., 2004). Using univariate Cox regression, the associations between overall survival (OS) and the lncRNAs, miRNAs, and mRNAs in the ceRNA network were assessed. Genes with a *P* < 0.05 in the univariate Cox regression analysis were included into the stepwise multivariate Cox regression analysis. The inclusion of these values generated a formula of the risk score. Ultimately, utilizing the median of the risk scores of lncRNA, miRNA, and mRNA as the threshold, we classified the risk into high and low to group the patients accordingly. We assessed the variations in OS among the patients who are grouped as low and high risk through the log-rank tests and Kaplan–Meier survival plots. Additionally, we adopted the analysis of the receiver operating characteristic (ROC) curve, along with the area under the ROC curve (AUC) to evaluate the survival prediction.

### Construction of Nomogram

**FIGURE 1:**
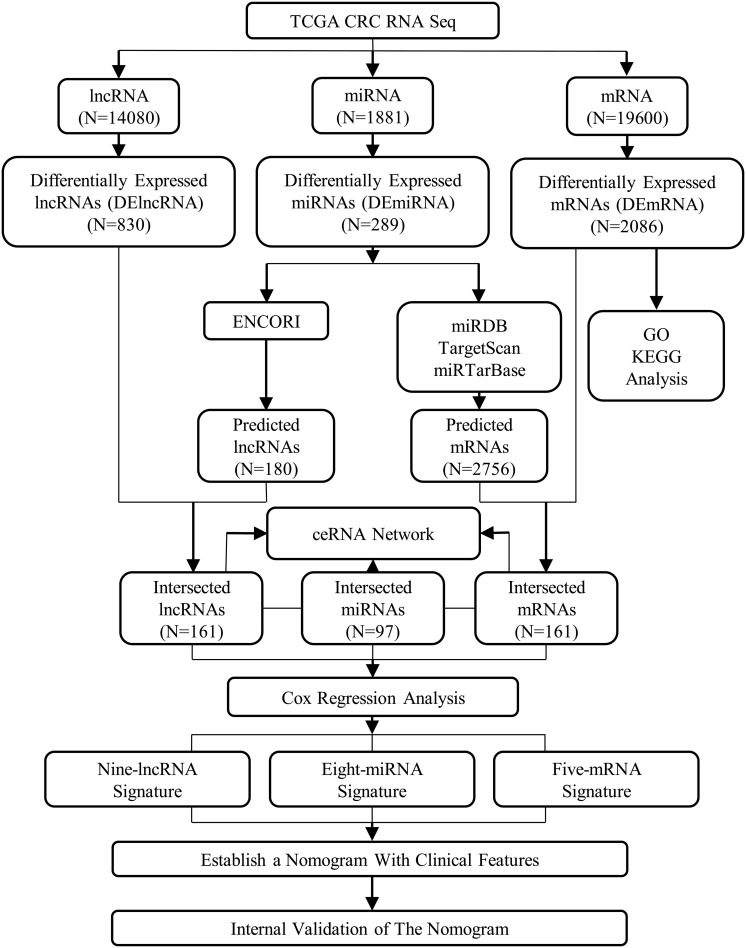
The flowchart of the designs and details of the lncRNA–miRNA–mRNA ceRNA network analysis.

There were 335 patients included in the nomogram, and the whole enrolled cohort was considered as the primary cohort. Fifty percent, 70%, and 90% of patients from the primary cohort were randomly selected as validation sets. An assessment was conducted on the associations of relevant clinical features and ceRNAs risk score with OS using Cox proportional hazards regression models. For the multivariable Cox proportional hazards regression models, variables were identified using both the Akaike Information Criterion (AIC) and the concordance index (C-index). To predict the probability of 1-, 3-, and 5-year OS, selected variables were incorporated in the nomogram using the statistical software, known as the rms in R, version 3.0.3 (Wang et al., 2013). Two indicators, namely the decision curve analysis (DCA) and the calibration curve, measured its predictive performance. The designs and details of the lncRNA–miRNA–mRNA ceRNA network analysis are displayed in the flowchart of Figure 1. Using the SPSS 21.0 statistical package (IBM Corporation, Armonk, NY, United States) and R project version 3.5.1^8^ for Windows, we performed all the statistical analyses and random allocation. Finally, we set the *P* < 0.05 to determine the statistical significance of the difference.

## Results

### Identifying DElncRNAs, DEmiRNAs, and DEmRNAs

**FIGURE 2:**
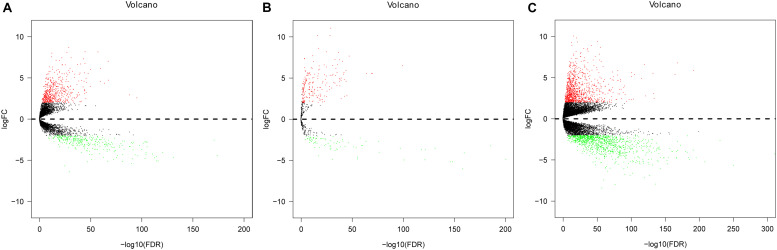
Volcano plot of differentially expressed lncRNAs (DElncRNAs), differentially expressed miRNAs (DEmiRNAs), and differentially expressed mRNAs (DEmRNAs) in colorectal cancer (CRC) and non-tumorous adjacent-normal samples. **(A)** Volcano plot of differentially expressed lncRNAs (DElncRNAs). **(B)** Volcano plot of differentially expressed miRNAs (DEmiRNAs). **(C)** Volcano plot of differentially expressed mRNAs (DEmRNAs). Log_2_fold change (log_2_FC) > 2 was labeled in red; log_2_FC < -2 was in green (*P* < 0.01).

Expression data of RNAs and miRNAs were collected from 386 specimens, which were composed of 335 CRC samples and 51 normal samples. After the RNAseq data were mapped to the human reference genome, we obtained a total of 14,080 lncRNAs, 1,881 miRNAs, and 19,600 mRNAs. By comparing the CRC group with the normal group, we identified the significant DElncRNAs, DEmiRNAs, and DEmRNAs using the edgeR package in R software. As a result, 830 DElncRNAs, 289 DEmiRNAs, and 2,086 DEmRNAs were confirmed by the edgeR package in R software. Of these, we inferred 529 upregulated DElncRNAs and 301 downregulated differential lncRNAs, 208 upregulated differential miRNAs and 81 downregulated miRNAs, and 1,041 upregulated differential mRNAs and 1,045 downregulated differential mRNAs, respectively (Figure 2).

### Functional Annotation

**FIGURE 3:**
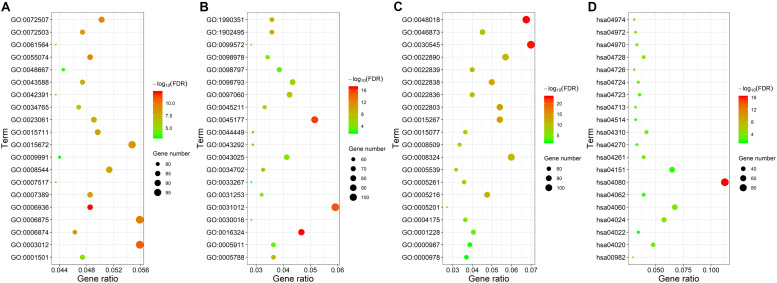
Gene ontology and KEGG pathway functional enrichment analyses of the differentially expressed mRNAs (DEmRNAs). **(A)** The biological function category of GO classification. **(B)** The cell component category of GO classification. **(C)** The molecular function category of GO classification. **(D)** KEGG pathway functional classification and annotation.

We analyzed these mRNAs using the R software package clusterprofiler to identify the functions linked to the DEmRNAs. This evaluation revealed the enrichment of 921 GO terms along with 55 KEGG pathways (FDR < 0.05). We chose to show the top 20 GO terms and 20 KEGG pathways of the DEmRNAs based on the gene count (Figure 3 and Supplementary Table S1). Biological processes of GO analyses were suggested to focus on muscle contraction (GO:0006936) and muscle system process (GO:0003012). The cellular component process found that the target genes are mainly clustered into the apical plasma membrane (GO:0016324) and apical part of cell (GO:0045177). Regarding the molecular functions process, the target genes are significantly related to receptor ligand activity (GO:0048018) and receptor regulator activity (GO:0030545). The KEGG pathways showed significant enrichment of the following pathways, such as neuroactive ligand–receptor interaction (hsa04080), drug metabolism–cytochrome P450 (hsa00982), salivary secretion (hsa04970), and cAMP signaling pathway (hsa04024).

### Construction of a ceRNA Network

**FIGURE 4:**
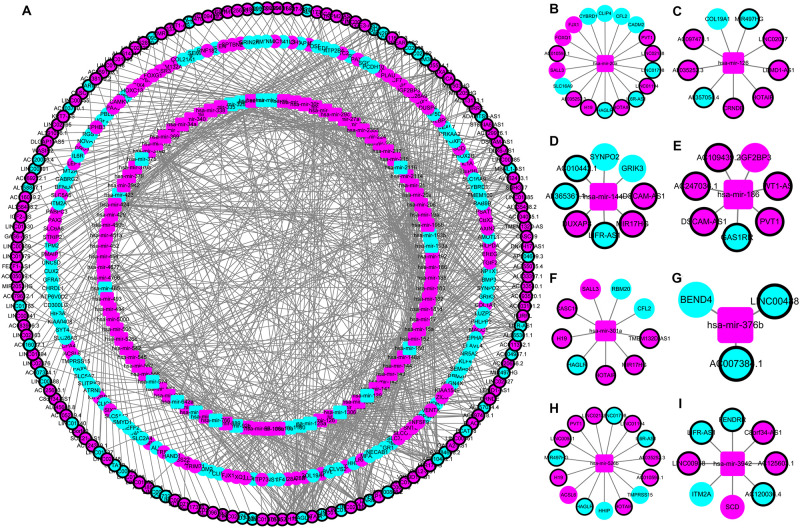
The lncRNA–miRNA–mRNA ceRNA network in colorectal cancer (CRC). **(A)** A global view of the ceRNA network in CRC. **(B–I)** hsa-mir-20a, hsa-mir-126, hsa-mir-144, hsa-mir-186, hsa-mir-301a, hsa-mir-376b, hsa-mir-526b, and hsa-mir-3942 mediated lncRNA-mRNA ceRNA network.

The construction of a ceRNA network graph that is built on these data provides comprehensive information on how lncRNA mediates mRNA through the combination of miRNA in CRC. Cytoscape v 3.7.0 provides a visual overview (Figure 4). We investigated the interactions between the DEmiRNAs and the DElncRNAs based on ENCORI, and 661 interactions between the DEmiRNAs and the DElncRNAs were predicted, which include 161 lncRNAs and 97 miRNAs. Next, the miRNA–mRNA target regulation network was further assessed using TargetScan, miRDB, and miRTarBase. Moving forward, these 97 miRNAs were found to interact with the 161 of the DEmRNAs. Ultimately, we selected 161 DElncRNAs, 97 DEmiRNAs, and 161 DEmRNAs for the construction of the ceRNA network (Supplementary Table S2).

### Survival Analysis for the ceRNAs

**FIGURE 5:**
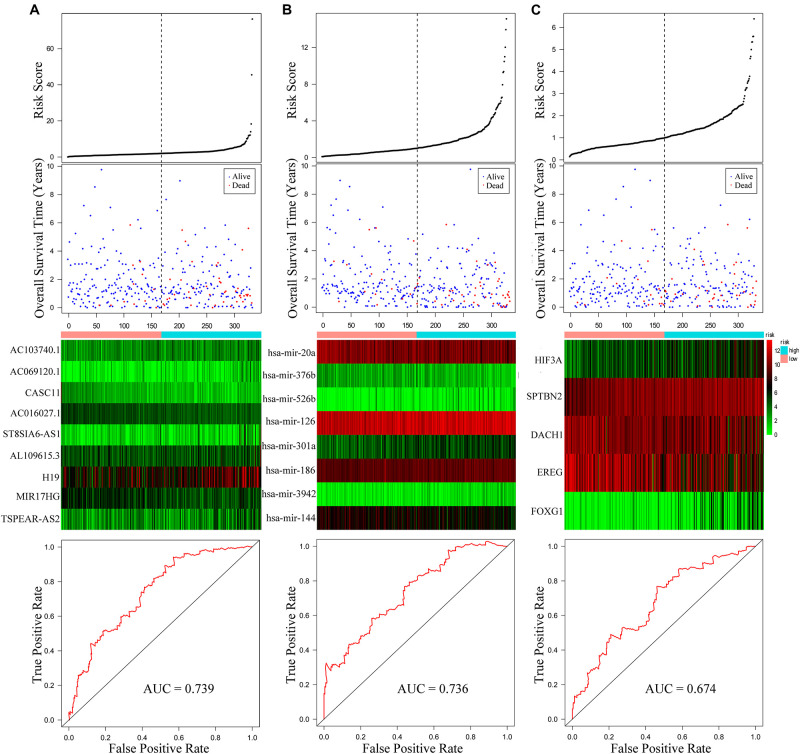
Risk score analysis of the nine-lncRNA, eight-miRNA, and five-mRNA signatures of CRC. **(A)** Risk score analysis, receiver operating characteristic (ROC) curve analysis, and risk heatmap of the nine-lncRNA signature expression profiles. **(B)** Risk score analysis, ROC curve analysis, and risk heatmap of the eight-miRNA signature expression profiles. **(C)** Risk score analysis, ROC curve analysis, and risk heatmap of the five-mRNA signature expression profiles.

**FIGURE 6:**
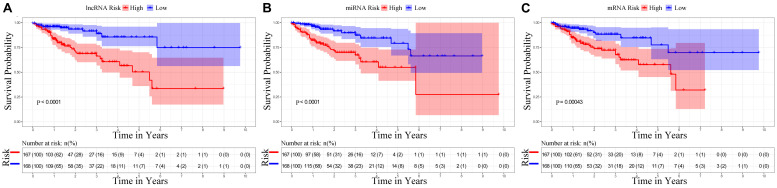
Kaplan–Meier survival curves for the nine lncRNAs, eight miRNAs, and five mRNAs in the ceRNA network related to overall survival. **(A)** Kaplan–Meier survival curves for the nine lncRNAs. **(B)** Kaplan–Meier survival curves for the eight miRNAs. **(C)** Kaplan–Meier survival curves for the five mRNAs.

The univariate Cox regression identified the significant prognostic value of 14 lncRNAs (Supplementary Table S3), 11 miRNAs (Supplementary Table S4), and seven mRNAs (Supplementary Table S5) in the ceRNA network. Using a stepwise multivariate Cox proportional hazards regression analysis, we indicated that nine-lncRNA (AC103740.1, AC069120.1, CASC11, AC016027.1, ST8SIA6-AS1, AL109615.3, H19, MIR17HG, and TSPEAR-AS2) signatures, eight-miRNA (hsa-mir-20a, hsa-mir-376b, hsa-mir-526b, hsa-mir-126, hsa-mir-301a, hsa-mir-186, hsa-mir-3942, and hsa-mir-144) signatures, and five-mRNA (HIF3A, SPTBN2, DACH1, EREG, and FOXG1) signatures showed a significant prognostic value for CRC. Figure 5 presents the risk scores and survival status of patients’ distribution in these three ceRNAs. The risk score model attained the AUC values of 0.739, 0.736, and 0.674 from the nine-lncRNA, eight-miRNA, and five-mRNA signatures accordingly. More specifically, the positive correlation of low risk with OS (*P* < 0.001, Figure 6) was confirmed by the Kaplan–Meier curves of these three signatures.

**FIGURE 7:**
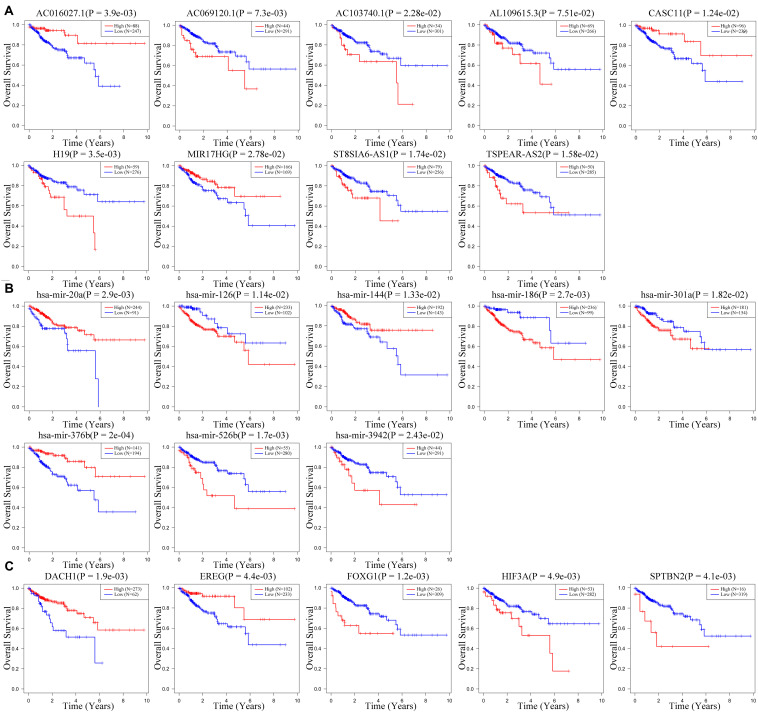
Kaplan–Meier survival curves for the nine-lncRNA, eight-miRNA, and five-mRNA signatures. **(A)** Kaplan–Meier survival curves for the nine-lncRNA signature. **(B)** Kaplan–Meier survival curves for the eight-miRNA signature. **(C)** Kaplan–Meier survival curves for the five-mRNA signature.

To further clarify their expression and prognostic value, we performed Kaplan–Meier survival analysis for the lncRNAs, miRNAs, and mRNAs in these three RNA signatures in patients with CRC from TCGA (Figure 7). The results showed that six lncRNAs (AC069120.1, AC103740.1, AL109615.3, H19, ST8SIA6-AS1, and TSPEAR-AS2), three mRNAs (FOXG1, HIF3A, and SPTBN2), and five miRNAs (hsa-mir-126, hsa-mir-186, hsa-mir-301a, hsa-mir-526b, and hsa-mir-3942) were identified as cancer-promoting factors because of their high expression correlation with shorter OS in patients with CRC. In contrast, another three lncRNAs (AC016027.1, CASC11, and MIR17HG), two mRNAs (DACH1, and EREG), and three miRNAs (hsa-mir-20a, hsa-mir-144, and hsa-mir-376b) showed high expression correlated with longer OS, implying that these RNAs might be protective factors in CRC.

### Establishment and Internal Validation of the Nomogram

**FIGURE 8:**
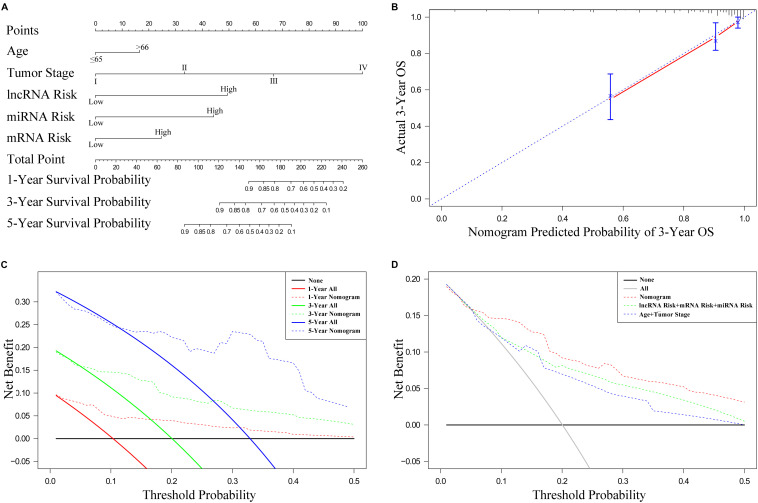
Nomogram, calibration plots, and decision curve analysis for the prediction of overall survival (OS) in patients with CRC. **(A)** Nomogram predicting 1-, 3-, and 5-year OS for CRC patients. **(B)** Calibration plot for nomogram predicted and observed 3-year overall survival rate. **(C)** Decision curve analysis for 1-, 3-, and 5-year overall survival predictions. **(D)** Decision curve analysis comparing nomogram with the lncRNA risk score + miRNA risk score + mRNA risk score model and the age + tumor stage.

To establish the nomogram, we evaluated the prognostic value of distinct clinical features. Table 1 demonstrates the correlation between eight of 13 evaluated clinical features and OS, which was verified through the univariate Cox proportional hazards regression. The independent prognostic indicators of CRC include the age, N stage, M stage, lncRNA risk score, and miRNA risk score. The smallest AIC value and highest C-index occurred when we incorporated five variables (age, tumor stage, lncRNA risk score, miRNA risk score, and mRNA risk score) into the multivariate Cox regression model for OS [AIC = 462.82, C-index = 0.838 (95% CI, 0.785–0.891)]. Figure 8A illustrates how nomogram predicted the OS of the CRC patients in the TCGA database. We predicted the survival probabilities of the CRC patients in three different timelines, at 1, 3, and 5 years using the nomogram. Figure 8B shows the calibration plot for the 3-year prediction of OS, which demonstrated a good overall prediction. For the estimation of OS risk, the nomogram displayed C-index of 0.822 (95% CI = 0.749–0.895), 0.845 (95% CI = 0.816–0.903), and 0.842 (95% CI = 0.787–0.897) in the 50, 70, and 90% validation cohort, respectively. As Figure 8C illustrates, the nomogram demonstrated a better net benefit that had a wider range of threshold probability on the DCA for predicting the corresponding 1-, 3-, and 5-year OS. Figure 8D represents how the DCA confirmed the higher net clinical benefit of the nomogram in contrast with the lncRNA risk score + miRNA risk score + mRNA risk score and age + tumor stage. This analysis took into account a wide selection of threshold probabilities that could predict the 3-year OS. Furthermore, at higher threshold probability levels, this is a representation of an exceptional estimation of decision outcomes.

**TABLE 1:** Cox proportional hazards regression model showing the association of variables with overall survival.

Variables		Patient *N* = 335	Univariate analysis			Multivariate analysis		
					
			HR	95% CI	*P*	HR	95% CI	*P*
Age	≤65	152	Reference			Reference		
	>65	183	1.815	1.010–3.261	0.046	1.944	1.074–3.518	0.028
Gender	Male	162	Reference					
	Female	173	1.013	0.775–1.324	0.924			
Tumor location	Right	147	Reference					
	Left	188	0.967	0.566–1.652	0.903			
Tumor recurrence	No	261	Reference					
	Yes	74	0.740	0.348–1.572	0.434			
Neoplasm cancer	Tumor free	153	Reference					
	With tumor	182	0.924	0.541–1.578	0.771			
Residual tumor	R0	280	Reference					
	R1 + R2	55	2.948	1.693- 5.133	<0.001			
Tumor stage	I	58	Reference					
	II	125	1.209	0.331–4.410	0.774			
	III	102	3.149	0.932–10.647	0.065			
	IV	50	7.806	2.325–26.207	0.001			
T stage	T1	12	Reference					
	T2	57	0.644	0.067–6.192	0.703			
	T3	233	1.827	0.250–13.320	0.522			
	T4	33	4.379	0.554–37.600	0.161			
N stage	N0	188	Reference			Reference		
	N1	88	2.498	1.259–4.958	0.009	2.771	1.331–5.766	0.006
	N2	59	4.923	2.522–9.609	<0.001	4.116	1.960–8.643	<0.001
M stage	M0	285	Reference			Reference		
	M1	50	4.178	2.413–7.234	<0.001	3.280	1.791–6.005	<0.001
lncRNA risk	Low	168	Reference			Reference		
	High	167	4.004	2.100–7.633	<0.001	4.900	2.485–9.662	<0.001
miRNA risk	Low	168	Reference			Reference		
	High	167	1.853	1.370–2.506	<0.001	4.027	2.155–7.524	<0.001
mRNA risk	Low	168	Reference					
	High	167	1.670	1.239–2.251	0.001			

*HR, hazard ratio; CI, confidence interval.*

**FIGURE 9:**
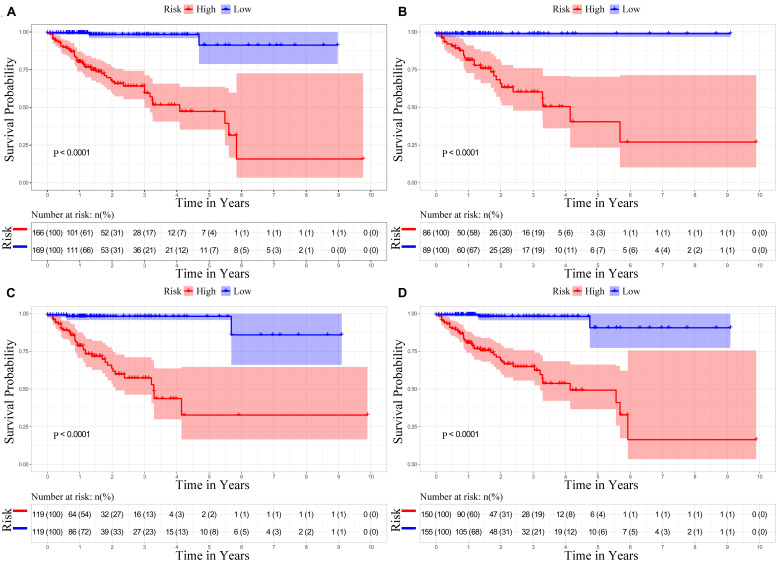
Kaplan–Meier analysis according to nomogram total points (NTP). **(A)** Kaplan–Meier survival curves for the primary cohort. **(B)** Kaplan–Meier survival curves for the 50% validation cohort. **(C)** Kaplan–Meier survival curves for the 70% validation cohort. **(D)** Kaplan–Meier survival curves for the 90% validation cohort.

To provide more plausible classifications of OS, patients were stratified into two groups that were based on an NTP’s optimal cutoff value identified by the median. Based on Figure 9A, the results suggest that patients from the high-risk group had significantly worse outcomes compared to the low-risk group (*P* < 0.001). Using the cohorts for validation, we verified the same results, as demonstrated by Figures 9B–D.

## Discussion

Globally, CRC is known for being one of the most frequently occurring malignancies, ranking second in women and third in men (Ferlay et al., 2015). Although great progress in the treatment, prognosis, and diagnosis of CRC, its mortality still remains high. The further study of efficient biomarkers and underlying mechanisms will drive the development of CRC diagnosis and treatment. We explored the CRC’s pathogenic mechanism in our study and identified a new prognostic predictor model for it.

The interaction of different RNA transcripts through shared MREs has been introduced in recent years through what is known as the ceRNA hypothesis (Salmena et al., 2011). Competitors that bind the common miRNA have been identified in many human ceRNAs in RNA regulatory networks (Denzler et al., 2014; Tay et al., 2014). Using the TCGA database, we utilized a broad cohort in this study to identify DElncRNAs, DEmiRNAs, and DEmRNAs between tissues that are normal and are of CRC. Eventually, we obtained a cohesive view of the ceRNA regulatory crosstalk between the RNA transcripts of these CRC-specific through the construction of the lncRNA–miRNA–mRNA ceRNA network. Many studies have been focused on the roles of lncRNAs in cancer initiation and progression (Iguchi et al., 2015; Raveh et al., 2015). In the ceRNA network, lncRNAs, miRNAs, and mRNAs are significantly linked to the CRC patients’ survival and clinical features. This study analyzes the regulatory interaction between RNAs that are non-coding and protein-coding. The results attempt to primarily define the ceRNA-mediated molecular mechanism that operates in the colorectal tumorigenesis at the level of transcription.

In various cancers, the evident presence of dysregulated lncRNAs demonstrated an association with patient survival (Gao et al., 2017; Sun et al., 2017; Zhang et al., 2017). Furthermore, the development of numerous lncRNA signatures improves the prognosis prediction of cancers that includes glioblastoma multiforme (Zhang et al., 2013), breast cancer (Meng et al., 2014), and lung cancer (Zhou et al., 2015). Recently, Wang et al. (2018) and Fan and Liu (2018) identified lncRNA signatures to prognosis prediction of CRC using Cox regression analysis. According to the expression values of nine DElncRNAs in the ceRNA network of patients, we generated a risk score model. We also found that, based on varied expression profiles of patients, nine-lncRNA signature differentiates CRC patients between poor and good prognoses. The nine-lncRNA signature is as follows: AC103740.1, AC069120.1, CASC11, AC016027.1, ST8SIA6-AS1, AL109615.3, H19, MIR17HG, and TSPEAR-AS2. A potential prognostic lncRNA, known as the ST8SIA6-AS1 from the nine lncRNAs, is recommended to become a tumor progression indicator that is applicable in different types of cancers (Fang et al., 2019; Luo et al., 2019). In the context of CRC, our findings confirmed ST8SIA6-AS1’s important role in CRC’s progression and supported existing reports by presenting consistent results. Nevertheless, more experimental evidence is required to verify the role of ST8SIA6-AS1 in the development of CRC.

In the instances where the components of various RNA transcripts affect each other, the miRNAs presented their key roles as the hub elements of the ceRNA network. Mediated by hsa-mir-301a, CASC19, H19, HAGLR, HOTAIR, MIR17HG, and TMEM132D-AS1 interacted with CFL2, RBM20, and SALL3 in the present CRC-specific ceRNA network. Consistent with our results, hsa-mir-301a was demonstrated to be involved in the pathogenesis of CRC, and high expression of hsa-mir-301a indicated an unfavorable clinical outcome (Zhang et al., 2014, 2019). Hence, we combined eight miRNAs, namely hsa-mir-20a, hsa-mir-376b, hsa-mir-526b, hsa-mir-126, hsa-mir-301a, hsa-mir-186, hsa-mir-3942, and hsa-mir-144, to develop a risk score. From this, we deduced the independent prediction of OS by this eight-miRNA signature in CRC patients. From our understanding, our study is the first that combines a ceRNA network with TCGA data and constructs a miRNA-related risk score to evaluate the OS of CRC patients.

An association was also found between some mRNAs in the ceRNA network and the survival of CRC patients, which is similar to lncRNAs and miRNAs. These mRNAs are the protective mRNA, such as DACH1 and EREG, and the risky mRNA, such as FOXG1, HIF3A, and SPTBN2. In many studies, the DACH1 has been involved in the prognosis of CRC (Yan et al., 2013; Wang, 2015). More specifically, reports showed that loss of DACH1 expression results in increased CRC cell growth, motility, and invasiveness through transforming growth factor β-mediated Epithelial-Mesenchymal Transition (EMT), and downregulation of DACH1 has important therapeutic implications for targeted therapies of CRC (Wang, 2015). In our ceRNA network, DACH1 could interact with hsa-mir-217, and many lncRNAs such as HOTAIR, LINC01748, and MIR17HG could function as molecular sponge to regulate hsa-mir-217. However, these lncRNA–miRNA–mRNA novel potential interaction hypotheses require further investigation and experimental confirmation. The establishment of a five-mRNA signature-based prognostic prediction system based on the mRNAs in the ceRNA network includes HIF3A, SPTBN2, DACH1, EREG, and FOXG1. As presented by the results of the Kaplan–Meier survival analysis, this system classified patients in the TCGA dataset into good, bad, and significantly different prognoses. In a univariable Cox regression model, we found a significantly shorter OS for patients with high-risk scores. This directly corresponds to an increase in CRC-related deaths. Overall, these results suggest that, in molecular pathogenesis, progression, and the prognosis of CRC, these mRNAs could play a fundamental role.

As far as we know, this is the first nomogram to combine clinicopathological data with lncRNA, miRNA, and mRNA signatures. In predicting the OS of CRC patients, the sufficient clarity and adequate calibration of the nomogram’s C-index were at 0.838. To further investigate the predictive ability of the nomogram, we reviewed some published nomograms for prognosis prediction in CRC, which were contained RNA signatures and clinical features (Jacob et al., 2018; Xiong et al., 2018; Huang et al., 2019; Lee et al., 2019; Mo et al., 2019; Song and Fu, 2019; Yang et al., 2020). We found that the C-index of our nomogram was significantly greater than that of other nomograms. Additionally, our recently completed nomogram model had a significantly higher level of net benefit, as demonstrated by the DCA results.

Despite our nomogram’s supportive role in the prediction of OS in CRC patients by the physicians, our research has several limitations. First, although the data of this study were all from the TCGA database, they were retrospective essentially, so a large-scale and multicenter prospective study should be launched to prove our results and eliminate the selective bias. Second, in our study, we used the mRNAseq, miRNAseq data, and clinicopathological data in the TCGA database to construct the nomogram. It is difficult to find a suitable similar dataset in other databases for external validation of this nomogram. However, the risk-prediction model presents optimal predictive power. Third, one final step still needs to be completed before clinical application. In large prospective trials, polymerase chain reaction–based validations would be of great significance in the clinical setting. Finally, to expound upon the inherent association between these RNA signatures and CRC prognosis, increase in experimental data on these RNAs is mandatory.

## Conclusion

In conclusion, with the use of bioinformatics analysis, our study revealed the CRC-specific ceRNA network. Using the information from the TCGA database, we also investigated their associations with clinical features. Furthermore, our research specifies that tumorigenesis occurs through the dysregulation of ceRNA network. We established a nomogram based on gene expression profile, clinical features, and pathological factors for predicting OS in CRC patients. Ultimately, the excellent discrimination and risk stratification of the formulated nomogram obtain the prediction OS in CRC patients.

## Data Availability Statement

Publicly available datasets were analyzed in this study. These data can be found in the Cancer Genome Atlas (https://tcga-data.nci.nih.gov/).

## Author Contributions

ZZ, YL, and XJia contributed to the planning of the study. WL and XJin conducted data analysis. WL, WY, GW, and XJia drafted the manuscript and revised the manuscript. XJia verified the numerical results by an independent implementation. WL, XJin, and GW prepared all the figures and tables. XJia, YL, and ZZ contributed to interpretation of data and review of the manuscript. All the authors reviewed and approved the final manuscript.

## Conflict of Interest

The authors declare that the research was conducted in the absence of any commercial or financial relationships that could be construed as a potential conflict of interest.

## Footnotes

1 https://tcga-data.nci.nih.gov/

2 http://www.ensembl.org/

3 http://cancergenome.nih.gov/publications/publicationguidelines

4 http://starbase.sysu.edu.cn/

5 http://www.mirdb.org/

6 http://www.targetscan.org/

7 http://mirtarbase.mbc.nctu.edu.tw/

8 http://www.r-project.org/
