# Supplementary material for: The Construction and Comprehensive Prognostic Analysis of the LncRNA-Associated Competitive Endogenous RNAs Network in Colorectal Cancer
Source: Front Genet. 2020 Jun 23;11:583. doi: 10.3389/fgene.2020.00583 (PMC7344331; doi:10.3389/fgene.2020.00583)
Supplement: Supplementary file 1 [file Table_1.DOCX]

**Table S1: Top 20 KEEG pathways and GO terms enriched by the DEmRNAs**

| Categories | Term | Description | Count | FDR |
| --- | --- | --- | --- | --- |
| KEGG pathways | hsa04080 | Neuroactive ligand-receptor interaction | 92 | 6.90E-17 |
|  | hsa04060 | Cytokine-cytokine receptor interaction | 55 | 0.000101 |
|  | hsa04151 | PI3K-Akt signaling pathway | 53 | 0.0191 |
|  | hsa04024 | cAMP signaling pathway | 47 | 1.70E-05 |
|  | hsa04020 | Calcium signaling pathway | 39 | 0.00029 |
|  | hsa04310 | Wnt signaling pathway | 34 | 0.000311 |
|  | hsa04728 | Dopaminergic synapse | 32 | 6.20E-05 |
|  | hsa04261 | Adrenergic signaling in cardiomyocytes | 32 | 0.000411 |
|  | hsa04062 | Chemokine signaling pathway | 32 | 0.019286 |
|  | hsa04723 | Retrograde endocannabinoid signaling | 29 | 0.00353 |
|  | hsa04724 | Glutamatergic synapse | 28 | 0.000113 |
|  | hsa04022 | cGMP-PKG signaling pathway | 28 | 0.033602 |
|  | hsa04970 | Salivary secretion | 27 | 8.98E-06 |
|  | hsa04270 | Vascular smooth muscle contraction | 27 | 0.003033 |
|  | hsa04713 | Circadian entrainment | 26 | 6.88E-05 |
|  | hsa04972 | Pancreatic secretion | 26 | 0.000113 |
|  | hsa04514 | Cell adhesion molecules (CAMs) | 26 | 0.023517 |
|  | hsa04974 | Protein digestion and absorption | 25 | 0.000101 |
|  | hsa04726 | Serotonergic synapse | 25 | 0.002015 |
|  | hsa00982 | Drug metabolism - cytochrome P450 | 24 | 7.96E-06 |
| GO BP | GO:0003012 | muscle system process | 99 | 1.67E-11 |
|  | GO:0006875 | cellular metal ion homeostasis | 99 | 1.94E-10 |
|  | GO:0015672 | monovalent inorganic cation transport | 97 | 1.86E-09 |
|  | GO:0008544 | epidermis development | 91 | 1.03E-08 |
|  | GO:0072507 | divalent inorganic cation homeostasis | 89 | 5.83E-10 |
|  | GO:0015711 | organic anion transport | 88 | 9.50E-09 |
|  | GO:0023061 | signal release | 87 | 2.35E-08 |
|  | GO:0006936 | muscle contraction | 86 | 6.16E-13 |
|  | GO:0055074 | calcium ion homeostasis | 86 | 4.40E-10 |
|  | GO:0007389 | pattern specification process | 86 | 3.68E-09 |
|  | GO:0072503 | cellular divalent inorganic cation homeostasis | 84 | 2.84E-09 |
|  | GO:0043588 | skin development | 84 | 2.35E-08 |
|  | GO:0001501 | skeletal system development | 84 | 9.27E-06 |
|  | GO:0034765 | regulation of ion transmembrane transport | 83 | 2.34E-06 |
|  | GO:0006874 | cellular calcium ion homeostasis | 82 | 1.86E-09 |
|  | GO:0048667 | cell morphogenesis involved in neuron differentiation | 79 | 0.000475 |
|  | GO:0009991 | response to extracellular stimulus | 78 | 0.000684 |
|  | GO:0042391 | regulation of membrane potential | 77 | 6.55E-08 |
|  | GO:0007517 | muscle organ development | 77 | 1.45E-06 |
|  | GO:0061564 | axon development | 77 | 2.06E-05 |
| GO CC | GO:0031012 | extracellular matrix | 109 | 1.03E-15 |
|  | GO:0045177 | apical part of cell | 95 | 8.47E-17 |
|  | GO:0016324 | apical plasma membrane | 86 | 1.08E-17 |
|  | GO:0098793 | presynapse | 80 | 1.02E-06 |
|  | GO:0097060 | synaptic membrane | 78 | 5.05E-08 |
|  | GO:0043025 | neuronal cell body | 76 | 4.40E-05 |
|  | GO:0098797 | plasma membrane protein complex | 71 | 0.000565 |
|  | GO:0005788 | endoplasmic reticulum lumen | 67 | 9.42E-09 |
|  | GO:0005911 | cell-cell junction | 67 | 0.00041 |
|  | GO:1902495 | transmembrane transporter complex | 66 | 2.26E-10 |
|  | GO:1990351 | transporter complex | 66 | 4.29E-10 |
|  | GO:0098978 | glutamatergic synapse | 63 | 3.42E-05 |
|  | GO:0045211 | postsynaptic membrane | 61 | 7.74E-07 |
|  | GO:0034702 | ion channel complex | 60 | 2.12E-09 |
|  | GO:0031253 | cell projection membrane | 59 | 2.30E-05 |
|  | GO:0044449 | contractile fiber part | 53 | 2.26E-10 |
|  | GO:0043292 | contractile fiber | 53 | 3.49E-09 |
|  | GO:0030016 | myofibril | 52 | 1.29E-09 |
|  | GO:0099572 | postsynaptic specialization | 52 | 0.001633 |
|  | GO:0033267 | axon part | 52 | 0.014046 |
| GO MF | GO:0030545 | receptor regulator activity | 119 | 1.29E-23 |
|  | GO:0048018 | receptor ligand activity | 115 | 7.29E-24 |
|  | GO:0008324 | cation transmembrane transporter activity | 102 | 2.08E-11 |
|  | GO:0022890 | inorganic cation transmembrane transporter activity | 97 | 9.56E-12 |
|  | GO:0015267 | channel activity | 92 | 2.01E-14 |
|  | GO:0022803 | passive transmembrane transporter activity | 92 | 2.01E-14 |
|  | GO:0022838 | substrate-specific channel activity | 85 | 9.99E-14 |
|  | GO:0005216 | ion channel activity | 81 | 1.47E-12 |
|  | GO:0046873 | metal ion transmembrane transporter activity | 77 | 2.05E-10 |
|  | GO:0001228 | transcriptional activator activity, RNA polymerase II transcription regulatory region sequence-specific DNA binding | 69 | 4.34E-05 |
|  | GO:0022839 | ion gated channel activity | 68 | 2.21E-11 |
|  | GO:0022836 | gated channel activity | 68 | 2.43E-11 |
|  | GO:0000987 | proximal promoter sequence-specific DNA binding | 66 | 0.011421 |
|  | GO:0000978 | RNA polymerase II proximal promoter sequence-specific DNA binding | 63 | 0.014699 |
|  | GO:0015077 | monovalent inorganic cation transmembrane transporter activity | 62 | 7.63E-09 |
|  | GO:0004175 | endopeptidase activity | 62 | 8.22E-06 |
|  | GO:0005261 | cation channel activity | 61 | 3.29E-09 |
|  | GO:0008509 | anion transmembrane transporter activity | 57 | 1.77E-08 |
|  | GO:0005539 | glycosaminoglycan binding | 54 | 3.41E-10 |
|  | GO:0005201 | extracellular matrix structural constituent | 46 | 7.91E-10 |
