# Supplementary material for: The Construction and Comprehensive Prognostic Analysis of the LncRNA-Associated Competitive Endogenous RNAs Network in Colorectal Cancer
Source: Front Genet. 2020 Jun 23;11:583. doi: 10.3389/fgene.2020.00583 (PMC7344331; doi:10.3389/fgene.2020.00583)
Supplement: Supplementary file 2 [file Table_2.DOCX]

**Table S2:** **Selected DElncRNAs, DEmiRNAs, and DEmRNAs for the construction of the ceRNA network**

| lncRNA | logFC | FDR | lncRNA | logFC | FDR | miRNA | logFC | FDR | mRNA | logFC | FDR | mRNA | logFC | FDR |
| --- | --- | --- | --- | --- | --- | --- | --- | --- | --- | --- | --- | --- | --- | --- |
| CDKN2B-AS1 | -5.09 | 6.97E-117 | AC097478.1 | 3.06 | 3.09E-07 | hsa-mir-328 | -5.44 | 0 | BMP3 | -5.21 | 2.07E-118 | MYBL2 | 2.22 | 6.71E-45 |
| ADAMTS9-AS1 | -4.42 | 4.48E-97 | AC011611.3 | 3.07 | 4.23E-22 | hsa-mir-1224 | -4.90 | 8.20E-32 | NPTX1 | -5.16 | 3.88E-113 | DACH1 | 2.25 | 1.56E-25 |
| AC073283.2 | -4.03 | 4.59E-105 | G2E3-AS1 | 3.11 | 1.70E-05 | hsa-mir-139 | -4.87 | 2.27E-201 | CADM2 | -4.74 | 1.02E-81 | MYCN | 2.25 | 7.17E-18 |
| AC004947.1 | -3.42 | 3.37E-79 | GAS6-AS1 | 3.17 | 2.13E-31 | hsa-mir-766 | -4.09 | 1.40E-160 | PHOX2B | -4.65 | 2.26E-81 | MACC1 | 2.26 | 1.05E-51 |
| LINC00891 | -3.27 | 4.04E-67 | TRPM2-AS | 3.20 | 3.43E-26 | hsa-mir-1306 | -3.72 | 9.84E-123 | SMYD1 | -4.64 | 2.78E-62 | TP73 | 2.27 | 2.27E-24 |
| LINC00461 | -3.21 | 1.20E-48 | LINC01605 | 3.20 | 1.36E-46 | hsa-mir-574 | -3.70 | 7.99E-119 | PRIMA1 | -4.59 | 9.10E-83 | PABPC3 | 2.28 | 2.18E-21 |
| AC010442.1 | -3.19 | 1.12E-98 | AL513123.1 | 3.23 | 1.43E-22 | hsa-mir-642a | -3.69 | 1.75E-36 | SLC26A3 | -4.51 | 1.20E-84 | SNTB1 | 2.33 | 5.06E-52 |
| AP002358.1 | -3.08 | 1.06E-42 | AC124319.1 | 3.29 | 1.63E-29 | hsa-mir-6720 | -3.60 | 3.65E-30 | SYNM | -4.42 | 2.53E-94 | GRHL1 | 2.34 | 8.18E-40 |
| PCAT18 | -3.01 | 1.57E-27 | AC011352.1 | 3.30 | 2.51E-10 | hsa-mir-125a | -3.54 | 3.98E-132 | ADCYAP1R1 | -4.39 | 1.67E-83 | KIAA1549 | 2.38 | 6.95E-80 |
| MBNL1-AS1 | -2.97 | 1.08E-91 | LINC01485 | 3.32 | 4.63E-15 | hsa-mir-1180 | -3.37 | 1.47E-39 | SLC5A7 | -4.31 | 1.95E-60 | STRIP2 | 2.38 | 3.18E-43 |
| AC103740.1 | -2.93 | 2.56E-102 | C8orf49 | 3.35 | 8.85E-07 | hsa-mir-150 | -3.36 | 4.73E-52 | SFRP1 | -4.24 | 6.18E-80 | ARID3A | 2.42 | 8.96E-30 |
| LINC02568 | -2.92 | 1.87E-42 | LINC02027 | 3.37 | 3.15E-08 | hsa-mir-3173 | -3.35 | 2.30E-29 | TMEFF2 | -4.22 | 9.69E-92 | SCD | 2.42 | 1.60E-41 |
| CARMN | -2.90 | 1.18E-59 | HOTAIR | 3.38 | 2.46E-07 | hsa-mir-193a | -3.20 | 3.05E-61 | TRPM6 | -4.15 | 4.31E-123 | ITGBL1 | 2.45 | 2.03E-14 |
| GAS1RR | -2.88 | 2.70E-41 | AL033397.1 | 3.39 | 1.38E-09 | hsa-mir-326 | -2.88 | 4.34E-33 | HAND1 | -3.99 | 5.91E-30 | SLC7A11 | 2.47 | 4.06E-34 |
| FOXD3-AS1 | -2.87 | 2.77E-31 | DUXAP8 | 3.47 | 2.99E-21 | hsa-mir-92b | -2.86 | 1.84E-47 | SLC5A12 | -3.96 | 1.73E-57 | HILPDA | 2.48 | 1.14E-61 |
| AC020978.4 | -2.83 | 6.30E-91 | DNAH17-AS1 | 3.53 | 1.36E-22 | hsa-mir-485 | -2.83 | 2.69E-38 | SYNPO2 | -3.91 | 2.73E-85 | SIX4 | 2.57 | 4.03E-20 |
| AL357054.4 | -2.81 | 1.21E-92 | LINC01630 | 3.59 | 1.24E-08 | hsa-mir-193b | -2.66 | 9.24E-42 | TMEM100 | -3.88 | 7.24E-138 | AXIN2 | 2.64 | 8.27E-38 |
| SFTA1P | -2.78 | 4.09E-39 | LINC01978 | 3.60 | 3.43E-34 | hsa-mir-3150b | -2.42 | 5.98E-11 | KIAA0408 | -3.76 | 9.35E-73 | TMEM132A | 2.72 | 4.16E-72 |
| LINC01537 | -2.74 | 2.13E-58 | LINC01977 | 3.61 | 4.49E-45 | hsa-mir-375 | -2.33 | 8.45E-16 | EPHA7 | -3.69 | 1.05E-63 | IL1A | 2.73 | 1.36E-17 |
| MIR497HG | -2.74 | 4.92E-105 | AC020656.2 | 3.68 | 4.68E-25 | hsa-mir-423 | -2.28 | 1.04E-58 | SLITRK3 | -3.65 | 5.27E-36 | COL1A1 | 2.75 | 1.82E-28 |
| LINC01798 | -2.65 | 2.87E-40 | LINC02476 | 3.72 | 1.86E-06 | hsa-mir-2116 | -2.23 | 1.04E-12 | GRIK3 | -3.64 | 1.97E-100 | CELSR3 | 2.77 | 5.21E-44 |
| AL365361.1 | -2.64 | 1.05E-46 | IGF2-AS | 3.72 | 1.24E-11 | hsa-mir-185 | 2.06 | 1.22E-17 | CLVS2 | -3.52 | 3.23E-43 | PAX3 | 2.82 | 4.74E-07 |
| AP004609.3 | -2.62 | 5.37E-26 | LINC01748 | 3.80 | 4.46E-29 | hsa-mir-186 | 2.10 | 8.84E-22 | LIFR | -3.49 | 2.66E-140 | PAX9 | 2.82 | 4.10E-22 |
| AC016027.1 | -2.55 | 1.38E-171 | AC005089.1 | 3.90 | 1.69E-43 | hsa-mir-130a | 2.13 | 9.50E-12 | ELAVL4 | -3.43 | 4.98E-99 | PAX2 | 2.84 | 2.35E-09 |
| LIFR-AS1 | -2.51 | 3.40E-51 | AC105219.2 | 3.94 | 1.62E-28 | hsa-mir-493 | 2.20 | 1.52E-12 | NOVA1 | -3.38 | 6.06E-77 | PSAT1 | 2.90 | 7.77E-51 |
| LINC00402 | -2.49 | 7.74E-30 | H19 | 3.94 | 8.96E-18 | hsa-mir-30e | 2.48 | 4.12E-40 | SYT4 | -3.34 | 1.96E-33 | LRP8 | 2.94 | 4.72E-92 |
| IL6R-AS1 | -2.47 | 1.81E-59 | CASC19 | 3.96 | 1.87E-50 | hsa-mir-181d | 2.65 | 5.27E-07 | CHRDL1 | -3.27 | 7.17E-48 | HEPHL1 | 2.96 | 6.28E-12 |
| TARID | -2.42 | 9.01E-57 | LINC02188 | 3.98 | 5.37E-13 | hsa-mir-494 | 2.67 | 6.53E-06 | UNC5D | -3.22 | 2.07E-71 | EREG | 2.98 | 9.36E-18 |
| HAGLR | -2.35 | 2.26E-36 | PLAC4 | 3.99 | 2.07E-28 | hsa-mir-34a | 2.68 | 5.74E-17 | GFRA1 | -3.22 | 1.02E-77 | MEX3A | 2.99 | 2.67E-46 |
| LINC02245 | -2.34 | 3.53E-28 | DLX6-AS1 | 4.00 | 1.33E-14 | hsa-mir-26b | 2.74 | 1.27E-25 | ALK | -3.21 | 7.27E-51 | SLC6A6 | 2.99 | 1.07E-62 |
| FAM30A | -2.33 | 3.86E-27 | AL117329.1 | 4.08 | 4.38E-09 | hsa-mir-27a | 2.82 | 6.43E-24 | NEGR1 | -3.20 | 5.63E-123 | FJX1 | 3.10 | 1.94E-59 |
| AP000866.2 | -2.32 | 3.85E-74 | UCA1 | 4.13 | 4.72E-23 | hsa-mir-340 | 2.85 | 1.31E-15 | NECAB1 | -3.20 | 7.55E-75 | SPTBN2 | 3.20 | 2.93E-89 |
| LINC02292 | -2.30 | 7.52E-50 | AC109439.2 | 4.16 | 1.17E-07 | hsa-mir-31 | 2.92 | 0.00185428 | CD300LG | -3.11 | 1.82E-25 | COL7A1 | 3.31 | 6.73E-42 |
| LINC01783 | -2.29 | 1.30E-19 | AC129926.1 | 4.16 | 7.56E-16 | hsa-mir-372 | 3.03 | 0.04790548 | GABRG2 | -3.10 | 4.59E-25 | DUSP4 | 3.35 | 1.94E-25 |
| LINC00488 | -2.27 | 1.24E-14 | LINC00941 | 4.18 | 7.52E-23 | hsa-mir-224 | 3.07 | 1.92E-12 | ATRNL1 | -3.09 | 4.01E-49 | TNFSF9 | 3.42 | 1.56E-27 |
| AC007384.1 | -2.25 | 2.38E-53 | DSCAM-AS1 | 4.21 | 1.03E-06 | hsa-mir-338 | 3.09 | 7.79E-09 | SEMA6D | -3.03 | 2.00E-111 | SIX1 | 3.44 | 1.22E-25 |
| LINC01140 | -2.21 | 9.15E-52 | AC133785.1 | 4.29 | 1.60E-18 | hsa-mir-29c | 3.10 | 7.26E-14 | GRIN2A | -2.91 | 1.91E-37 | TGFBI | 3.46 | 2.25E-68 |
| FENDRR | -2.12 | 1.83E-57 | CRNDE | 4.36 | 2.08E-64 | hsa-mir-196b | 3.16 | 1.28E-08 | BVES | -2.89 | 2.75E-80 | FOXG1 | 3.51 | 2.62E-07 |
| AC120036.4 | -2.08 | 1.47E-25 | PURPL | 4.40 | 1.83E-12 | hsa-mir-33a | 3.35 | 9.87E-11 | PCDH10 | -2.88 | 3.19E-32 | SALL3 | 3.54 | 2.23E-06 |
| AL158847.1 | -2.04 | 2.74E-21 | AC010595.1 | 4.45 | 1.71E-09 | hsa-mir-223 | 3.37 | 1.99E-11 | RBM20 | -2.86 | 1.09E-55 | STC2 | 3.61 | 4.33E-40 |
| AC009549.1 | -2.01 | 1.40E-19 | AL109615.3 | 4.46 | 8.18E-47 | hsa-mir-451a | 3.38 | 3.31E-11 | TNS1 | -2.83 | 1.33E-67 | SIX3 | 3.63 | 4.19E-11 |
| BX284668.2 | -2.00 | 3.37E-18 | LINC02432 | 4.49 | 1.58E-12 | hsa-mir-30b | 3.41 | 2.75E-26 | COL19A1 | -2.82 | 2.40E-32 | RNF182 | 3.63 | 1.86E-15 |
| AC003101.2 | 2.00 | 4.82E-12 | SNHG25 | 4.57 | 1.00E-26 | hsa-mir-183 | 3.50 | 2.88E-17 | CHL1 | -2.76 | 1.09E-66 | LIN28A | 3.69 | 4.96E-08 |
| AC138150.2 | 2.03 | 4.30E-26 | AC007128.1 | 4.58 | 2.60E-60 | hsa-mir-143 | 3.56 | 1.06E-10 | BEND4 | -2.74 | 2.14E-55 | PRSS22 | 3.73 | 1.16E-56 |
| AL356488.2 | 2.07 | 3.33E-06 | AC104035.1 | 4.61 | 1.02E-16 | hsa-mir-526b | 3.73 | 0.03203268 | IL6R | -2.74 | 6.05E-169 | CPA4 | 3.74 | 4.22E-24 |
| AC093520.1 | 2.07 | 3.34E-10 | LINC00355 | 4.88 | 1.11E-09 | hsa-mir-17 | 3.79 | 6.43E-31 | WDR17 | -2.72 | 3.30E-31 | CBX2 | 3.78 | 9.58E-74 |
| AC069120.1 | 2.09 | 6.21E-06 | RMRP | 4.89 | 1.74E-07 | hsa-mir-3942 | 3.81 | 0.00077648 | SGK1 | -2.72 | 1.17E-115 | IGF2BP3 | 3.84 | 4.40E-15 |
| SNHG17 | 2.09 | 1.25E-40 | MIR205HG | 4.93 | 4.95E-07 | hsa-mir-2114 | 3.82 | 0.02544266 | CFL2 | -2.71 | 2.14E-95 | TRIB3 | 3.99 | 2.61E-91 |
| AL645608.8 | 2.09 | 9.30E-08 | AL161431.1 | 5.38 | 1.44E-28 | hsa-mir-192 | 3.84 | 4.62E-20 | EPB41L3 | -2.71 | 1.41E-73 | CAMKV | 4.22 | 1.02E-21 |
| AC005534.1 | 2.10 | 1.71E-18 | AC247036.1 | 5.39 | 6.56E-08 | hsa-mir-10a | 3.86 | 8.02E-17 | MFAP5 | -2.70 | 7.48E-43 | VENTX | 4.28 | 5.50E-19 |
| EIPR1-IT1 | 2.10 | 3.16E-12 | FIRRE | 5.44 | 1.12E-39 | hsa-mir-32 | 3.96 | 1.09E-28 | RGS13 | -2.70 | 2.17E-35 | DSC3 | 4.32 | 7.18E-19 |
| LUCAT1 | 2.12 | 1.96E-12 | AL355075.4 | 5.46 | 6.66E-09 | hsa-mir-4766 | 4.01 | 0.00050907 | TMPRSS15 | -2.69 | 6.69E-10 | ULBP2 | 4.36 | 6.41E-39 |
| PRR7-AS1 | 2.14 | 1.72E-40 | DLGAP1-AS5 | 5.49 | 4.93E-10 | hsa-mir-152 | 4.04 | 6.82E-39 | SLC2A4 | -2.66 | 2.29E-75 | ACSL6 | 4.48 | 3.55E-29 |
| AC069222.1 | 2.15 | 7.56E-20 | AC117386.2 | 5.58 | 2.27E-27 | hsa-mir-15a | 4.13 | 5.18E-36 | CUX2 | -2.65 | 5.85E-50 | LIN28B | 4.49 | 8.88E-07 |
| LINC00958 | 2.18 | 2.98E-07 | LINC00858 | 5.60 | 1.65E-40 | hsa-mir-369 | 4.15 | 9.50E-25 | CPM | -2.65 | 3.61E-99 | MMP11 | 4.58 | 1.26E-60 |
| AL035458.2 | 2.21 | 4.98E-21 | IGFL2-AS1 | 5.63 | 2.79E-20 | hsa-mir-5000 | 4.27 | 2.80E-09 | PHLPP2 | -2.63 | 1.88E-231 | CXCL5 | 4.73 | 3.20E-19 |
| LINC01641 | 2.21 | 3.99E-07 | AFAP1-AS1 | 5.93 | 1.75E-17 | hsa-mir-376b | 4.29 | 2.89E-08 | TPM2 | -2.59 | 1.40E-64 | HOXC13 | 4.81 | 1.40E-10 |
| MIR17HG | 2.25 | 1.80E-33 | LINC00659 | 6.03 | 1.17E-50 | hsa-mir-506 | 4.33 | 0.01773395 | RPH3A | -2.50 | 4.19E-35 | IGF2BP1 | 5.16 | 1.88E-17 |
| AC083906.3 | 2.28 | 3.25E-10 | AL162413.1 | 6.56 | 3.42E-23 | hsa-mir-656 | 4.35 | 3.27E-05 | THRB | -2.50 | 8.97E-51 | TRIM71 | 5.79 | 1.51E-17 |
| LINC00885 | 2.32 | 2.56E-08 | LINC01602 | 6.62 | 5.19E-23 | hsa-mir-148a | 4.38 | 4.41E-34 | KLF4 | -2.37 | 8.72E-85 | EN2 | 6.24 | 2.88E-24 |
| LINC00661 | 2.34 | 0.0022289 | LINC02163 | 7.02 | 4.46E-68 | hsa-mir-217 | 4.40 | 2.59E-12 | LUZP2 | -2.37 | 5.97E-40 | SLCO1B3 | 6.53 | 5.33E-32 |
| AC020891.2 | 2.40 | 3.10E-19 | TMEM132D-AS1 | 8.01 | 5.93E-13 | hsa-mir-106a | 4.46 | 7.81E-10 | SLC16A9 | -2.33 | 5.83E-46 | FOXQ1 | 6.62 | 1.50E-120 |
| AC105219.4 | 2.42 | 4.02E-32 | LINC02418 | 8.16 | 4.30E-58 | hsa-mir-429 | 4.49 | 6.31E-33 | IRF4 | -2.32 | 3.78E-40 | SLC26A9 | 6.92 | 4.20E-19 |
| LINC01433 | 2.42 | 5.02E-21 | FEZF1-AS1 | 8.17 | 1.99E-45 | hsa-mir-206 | 4.60 | 0.00375847 | NNAT | -2.32 | 1.25E-32 | ZIC5 | 7.91 | 1.47E-26 |
| CASC11 | 2.44 | 1.24E-10 |  |  |  | hsa-mir-4677 | 4.61 | 2.85E-22 | SEMA6A | -2.30 | 3.02E-54 |  |  |  |
| AC116049.2 | 2.45 | 2.52E-09 |  |  |  | hsa-mir-126 | 4.61 | 6.08E-45 | ATP2B4 | -2.28 | 3.11E-75 |  |  |  |
| LINC01194 | 2.46 | 0.00092141 |  |  |  | hsa-mir-2355 | 4.62 | 1.38E-38 | RAB9B | -2.27 | 5.55E-49 |  |  |  |
| LINC01694 | 2.46 | 6.56E-12 |  |  |  | hsa-mir-545 | 4.67 | 5.06E-06 | ITM2A | -2.24 | 8.82E-64 |  |  |  |
| LINC00922 | 2.49 | 4.10E-14 |  |  |  | hsa-mir-374b | 4.67 | 4.28E-40 | COL21A1 | -2.24 | 1.34E-45 |  |  |  |
| VPS9D1-AS1 | 2.53 | 3.12E-46 |  |  |  | hsa-mir-98 | 4.82 | 3.85E-43 | ATP6V0D2 | -2.24 | 1.73E-49 |  |  |  |
| WASIR2 | 2.55 | 1.07E-12 |  |  |  | hsa-mir-3613 | 4.85 | 1.36E-24 | NR5A2 | -2.23 | 1.03E-85 |  |  |  |
| PVT1 | 2.58 | 4.31E-96 |  |  |  | hsa-mir-301b | 4.91 | 9.72E-09 | OSR1 | -2.23 | 2.09E-25 |  |  |  |
| TSPEAR-AS2 | 2.60 | 1.26E-15 |  |  |  | hsa-mir-379 | 4.92 | 2.44E-38 | PBLD | -2.22 | 8.08E-79 |  |  |  |
| GATA2-AS1 | 2.61 | 1.41E-22 |  |  |  | hsa-mir-182 | 5.33 | 3.40E-31 | TCEAL7 | -2.21 | 3.36E-52 |  |  |  |
| AC018553.1 | 2.61 | 1.60E-07 |  |  |  | hsa-mir-708 | 5.33 | 1.98E-29 | KIF5C | -2.15 | 1.56E-38 |  |  |  |
| WT1-AS | 2.62 | 4.98E-07 |  |  |  | hsa-mir-556 | 5.34 | 2.96E-07 | CLIP4 | -2.15 | 4.55E-45 |  |  |  |
| AC104958.2 | 2.63 | 6.52E-35 |  |  |  | hsa-mir-96 | 5.40 | 8.39E-24 | MT2A | -2.15 | 4.53E-51 |  |  |  |
| AC133540.1 | 2.65 | 6.82E-12 |  |  |  | hsa-mir-452 | 5.46 | 4.22E-32 | HIF3A | -2.14 | 1.17E-27 |  |  |  |
| KRT7-AS | 2.69 | 7.97E-18 |  |  |  | hsa-mir-335 | 5.47 | 2.98E-42 | NLGN4X | -2.14 | 1.51E-40 |  |  |  |
| MIR2052HG | 2.74 | 1.17E-07 |  |  |  | hsa-mir-215 | 5.48 | 6.68E-14 | PAX5 | -2.12 | 2.22E-16 |  |  |  |
| AC125603.2 | 2.75 | 1.71E-11 |  |  |  | hsa-mir-301a | 5.51 | 8.96E-18 | EDIL3 | -2.11 | 4.76E-73 |  |  |  |
| AC125603.1 | 2.75 | 2.32E-07 |  |  |  | hsa-mir-141 | 5.57 | 6.12E-65 | UGP2 | -2.07 | 2.12E-183 |  |  |  |
| AC016831.5 | 2.80 | 3.26E-06 |  |  |  | hsa-mir-1277 | 5.58 | 4.17E-08 | HHIP | -2.05 | 9.68E-36 |  |  |  |
| LINC02195 | 2.82 | 1.44E-15 |  |  |  | hsa-mir-20a | 5.70 | 4.13E-36 | CYBRD1 | -2.04 | 4.62E-51 |  |  |  |
| AL662890.1 | 2.83 | 2.73E-19 |  |  |  | hsa-mir-542 | 5.93 | 6.86E-45 | CLIC5 | -2.04 | 9.54E-60 |  |  |  |
| LEMD1-AS1 | 2.85 | 1.76E-12 |  |  |  | hsa-mir-552 | 5.99 | 8.44E-18 | PRKAA2 | -2.02 | 2.11E-14 |  |  |  |
| SOX21-AS1 | 2.85 | 8.13E-06 |  |  |  | hsa-mir-424 | 6.19 | 5.78E-35 | AMOTL1 | -2.01 | 2.82E-41 |  |  |  |
| AL035252.3 | 2.87 | 2.62E-07 |  |  |  | hsa-mir-144 | 6.35 | 7.53E-26 | FOXF2 | -2.00 | 1.41E-50 |  |  |  |
| LINC00564 | 2.90 | 1.20E-05 |  |  |  | hsa-mir-628 | 6.48 | 1.53E-18 | TGIF2 | 2.00 | 4.86E-53 |  |  |  |
| C8orf34-AS1 | 2.91 | 4.87E-06 |  |  |  | hsa-mir-21 | 6.49 | 4.45E-100 | SLC5A6 | 2.03 | 1.49E-46 |  |  |  |
| LINC00491 | 2.97 | 7.90E-05 |  |  |  | hsa-mir-454 | 6.54 | 7.08E-32 | TCF7 | 2.07 | 1.82E-51 |  |  |  |
| MIR503HG | 2.97 | 3.06E-28 |  |  |  | hsa-mir-889 | 6.67 | 1.21E-36 | PMAIP1 | 2.09 | 6.36E-35 |  |  |  |
| LINC01979 | 3.00 | 1.20E-37 |  |  |  | hsa-mir-142 | 7.25 | 4.50E-34 | EPHB3 | 2.09 | 7.62E-24 |  |  |  |
| LINC02475 | 3.00 | 3.86E-07 |  |  |  | hsa-mir-450b | 8.19 | 1.45E-25 | RGS16 | 2.11 | 3.81E-39 |  |  |  |
| ST8SIA6-AS1 | 3.02 | 2.79E-07 |  |  |  | hsa-mir-577 | 8.61 | 3.76E-18 | ENC1 | 2.12 | 7.39E-94 |  |  |  |
| AL391056.1 | 3.02 | 1.84E-30 |  |  |  | hsa-mir-135b | 10.18 | 3.31E-29 | LEF1 | 2.15 | 3.41E-32 |  |  |  |
| AC079612.1 | 3.04 | 1.23E-16 |  |  |  | hsa-mir-19a | 11.06 | 6.25E-30 | PLAU | 2.18 | 1.66E-34 |  |  |  |

log FC: log Fold Change, FDR: False Discovery Rate.
