# Supplementary material for: The Construction and Comprehensive Prognostic Analysis of the LncRNA-Associated Competitive Endogenous RNAs Network in Colorectal Cancer
Source: Front Genet. 2020 Jun 23;11:583. doi: 10.3389/fgene.2020.00583 (PMC7344331; doi:10.3389/fgene.2020.00583)
Supplement: Supplementary file 3 [file Table_3.DOCX]

**Table S3: Prognostic value of the fourteen lncRNAs by cox regression analysis**

| lncRNA | HR | P Value | Coefficient |
| --- | --- | --- | --- |
| AC069120.1 | 1.020 | 0.023 | 3.098E-02 |
| AC103740.1 | 1.014 | 0.026 | 1.168E-02 |
| TSPEAR-AS2 | 1.005 | 0.038 | 4.177E-03 |
| ST8SIA6-AS1 | 1.003 | 0.013 | 3.223E-03 |
| AL109615.3 | 1.001 | 0.014 | 7.451E-04 |
| H19 | 1.000 | 0.038 | 2.859E-05 |
| MIR17HG | 0.995 | 0.046 | -5.799E-03 |
| AC016027.1 | 0.985 | 0.023 | -1.209E-02 |
| CASC11 | 0.929 | 0.040 | -7.424E-02 |
| AC020978.4 | 1.011 | 0.021 |  |
| FOXD3-AS1 | 1.029 | 0.022 |  |
| DLGAP1-AS5 | 1.001 | 0.031 |  |
| AP004609.3 | 1.128 | 0.034 |  |
| SOX21-AS1 | 1.007 | 0.044 |  |

HR: Hazard Ratio.
