# Supplementary material for: The Construction and Comprehensive Prognostic Analysis of the LncRNA-Associated Competitive Endogenous RNAs Network in Colorectal Cancer
Source: Front Genet. 2020 Jun 23;11:583. doi: 10.3389/fgene.2020.00583 (PMC7344331; doi:10.3389/fgene.2020.00583)
Supplement: Supplementary file 4 [file Table_4.DOCX]

**Table S4: Prognostic value of the eleven miRNAs by cox regression analysis**

| miRNA | HR | P Value | Coefficient |
| --- | --- | --- | --- |
| hsa-mir-20a | 0.745 | 0.040 | -0.493 |
| hsa-mir-376b | 0.740 | 0.015 | -0.490 |
| hsa-mir-144 | 0.789 | 0.048 | -0.322 |
| hsa-mir-301a | 1.350 | 0.016 | 0.216 |
| hsa-mir-526b | 1.292 | 0.027 | 0.361 |
| hsa-mir-126 | 1.450 | 0.049 | 0.435 |
| hsa-mir-186 | 1.792 | 0.018 | 0.472 |
| hsa-mir-3942 | 1.394 | 0.039 | 0.616 |
| hsa-mir-328 | 1.494 | 0.022 |  |
| hsa-mir-30e | 1.964 | 0.029 |  |
| hsa-mir-628 | 1.249 | 0.043 |  |

HR: Hazard Ratio.
