# Supplementary material for: The Construction and Comprehensive Prognostic Analysis of the LncRNA-Associated Competitive Endogenous RNAs Network in Colorectal Cancer
Source: Front Genet. 2020 Jun 23;11:583. doi: 10.3389/fgene.2020.00583 (PMC7344331; doi:10.3389/fgene.2020.00583)
Supplement: Supplementary file 5 [file Table_5.DOCX]

**Table S5: Prognostic value of the seven mRNAs by cox regression analysis**

| mRNA | HR | P Value | Coefficient |
| --- | --- | --- | --- |
| EREG | 0.893 | 0.031 | -0.122 |
| DACH1 | 0.850 | 0.024 | -0.120 |
| FOXG1 | 1.126 | 0.047 | 0.152 |
| HIF3A | 1.205 | 0.025 | 0.216 |
| SPTBN2 | 1.454 | 0.029 | 0.315 |
| PAX2 | 1.177 | 0.039 |  |
| ACSL6 | 0.908 | 0.041 |  |

HR: Hazard Ratio.
